# Detection of *Toxoplasma gondii* Infection in Small Ruminants: Old Problems, and Current Solutions

**DOI:** 10.3390/ani13172696

**Published:** 2023-08-23

**Authors:** Lucyna Holec-Gąsior, Karolina Sołowińska

**Affiliations:** Department of Molecular Biotechnology and Microbiology, Faculty of Chemistry, Gdańsk University of Technology, 11/12 Narutowicza Str., 80-233 Gdańsk, Poland; karolina.solowinska@pg.edu.pl

**Keywords:** *Toxoplasma gondii*, sheep, goat, toxoplasmosis, molecular detection, serological detection, recombinant antigen

## Abstract

**Simple Summary:**

Toxoplasmosis is a parasitic zoonosis of veterinary importance, with implications for public health. *Toxoplasma gondii* infection causes abortion or congenital disease in small ruminants. Moreover, the consumption of infected meat, cured meat products, or unpasteurized milk and dairy products can facilitate zoonotic transmission. This article presents the current status of the detection possibilities for *T. gondii* infection in small ruminants and their milk, focusing on molecular methods based on DNA amplification, including target genes employed in assays, and serological methods for the detection of specific anti-*T. gondii* antibodies with the use of recombinant antigens as single proteins, mixtures of antigens, or chimeric proteins consisting of fragments of various, properly selected *T. gondii* antigens.

**Abstract:**

Toxoplasmosis is a parasitic zoonosis of veterinary importance, with implications for public health. *Toxoplasma gondii* infection causes abortion or congenital disease in small ruminants. Moreover, the consumption of infected meat, cured meat products, or unpasteurized milk and dairy products can facilitate zoonotic transmission. Serological studies conducted in various European countries have shown the high seroprevalence of specific anti-*T. gondii* antibodies in sheep and goats related to the presence of oocysts in the environment, as well as climatic conditions. This article presents the current status of the detection possibilities for *T. gondii* infection in small ruminants and their milk. Serological testing is considered the most practical method for diagnosing toxoplasmosis; therefore, many studies have shown that recombinant antigens as single proteins, mixtures of various antigens, or chimeric proteins can be successfully used as an alternative to *Toxoplasma* lysate antigens (TLA). Several assays based on DNA amplification have been developed as alternative diagnostic methods, which are especially useful when serodiagnosis is not possible, e.g., the detection of intrauterine *T. gondii* infection when the fetus is not immunocompetent. These techniques employ multicopy sequences highly conserved among different strains of *T. gondii* in conventional, nested, competitive, and quantitative reverse transcriptase-PCR.

## 1. Introduction

*Toxoplasma gondii* is a widespread obligate, apicomplexan, protozoan parasite, responsible for toxoplasmosis in humans, livestock, and marine animals [1]. Toxoplasmosis was recognized as a parasitic zoonosis with a high incidence in humans by the European Food Safety Authority (EFSA) [2]. It is reported that about one-third of the adult human population is exposed to this parasite [3,4], and the prevalence of infection in different regions of the world varies from 0 to 100%, mainly in relation to climate, dietary and social habits, and socioeconomic levels [5]. Most *T. gondii* infections are clinically asymptomatic; however, this pathogen can cause severe disease in immunocompromised individuals and in congenitally infected fetuses of mothers who acquired primary infection during pregnancy. The risk and severity of fetal infection are linked to the moment of infection of a pregnant woman. While the risk increases with the duration of pregnancy, the severity of clinical symptoms declines over time. Thus, fetal infections may result in intrauterine death and spontaneous abortion, or in severe manifestations such as retinochoroiditis, hydrocephalus, intracranial calcifications, and fetal growth retardation [6]. Furthermore, primary *T. gondii* infection or the reactivation of chronic infection in immunosuppressed patients such as patients with AIDS is a major cause of encephalitis. 

Members of the family Felidae, including domestic cats, are the definitive host of *T. gondii*, which contaminates the environment with oocysts excreted with feces, while mammals and birds are intermediate hosts [5]. The *T. gondii* life cycle is perpetuated by the predatory nature of felines [7], which is confirmed by studies that report a significantly higher prevalence of anti-*T. gondii* antibodies in cats kept outdoors than indoors [8]. The predation of rodents, reptiles, insects, and birds commonly occurs and therefore constitutes the parasitic transmission route. Although *Toxoplasma* does not parasitize cold-blooded animals, their role as carriers of oocysts on the body surface or transiently in the guts has been reported [9,10,11] and plays a role in the chain of parasite transmission. Sporulated oocysts, generated after the sexual replication of the parasite in the intestinal tract of the definitive hosts, are responsible for the direct infection of humans and herbivores, birds, and rodents, usually through the contamination of vegetables, fruits, water, soil, and pastures. In intermediate hosts, *T. gondii* undergoes two phases of asexual development in which there are two morphological stages of the parasite, i.e., tachyzoite and bradyzoite. The early phase of primary infection is caused by tachyzoites capable of fast replication in many different types of host cells, which convert into latent chronic phase markers—slowly replicating bradyzoites contained in tissue cysts [12]. These tissue cysts remain in the brain and other organs (i.e., the eye, skeletal, and cardiac muscles) during the lifetime of infected intermediate hosts such as humans and domestic animals. Bradyzoites within tissue cysts are the source of infection transmitted from domestic animals to humans [5]. In meat-producing animals, tissue cysts of *T. gondii* are most frequently observed in the tissues of infected pigs, sheep, and goats; they are observed less frequently in infected poultry, rabbits, and horses [5]. Rarely, tissue cysts can be observed in cattle; however, there is no clear predilection site within bovine tissues [13]. Furthermore, in several hosts, tachyzoites may also be transmitted in the milk from the mother to the offspring [5,14]. By tachyzoites, the parasite may also be transmitted by blood transfusion, organ transplantation, or the consumption of unpasteurized goat or sheep dairy products [4,5]. However, more than 20 years ago, numerous studies reported that it is actually the preference to consume raw or undercooked meat that is the most important risk factor associated with human *T. gondii* infection [15,16,17]. It is noteworthy that small ruminant products are a major source of toxoplasmosis, mainly in those regions or countries where mutton and goat meat is routinely consumed or where the consumption of undercooked meat is a cultural and traditional habit [18]. In many countries, for example, lamb is often consumed undercooked and bradyzoites in tissue cysts are present in virtually all edible ovine tissues [1]. In addition, a large number of cysts were demonstrated not only in meat but also cured meat products from ovine or caprine hosts [16,18]. In 2020, Rani et al. [19] showed that even small serving sizes (5 g and 10 g) of goat and sheep meat have the potential for the transmission of *T. gondii* if consumed raw or undercooked. It is important to note that goats and sheep are highly susceptible to *T. gondii* infection. Numerous sources of epidemiological data show that antibodies to *T. gondii* have been found in small ruminants worldwide; however, the rate of seropositivity varies widely (ranging from 3% to 92%) and depends on the presence of oocysts in the environment and climatic conditions [5,20,21]. Serological studies conducted in various European countries have shown the high seroprevalence of specific anti-*T. gondii* antibodies in sheep and goats [22,23,24,25,26,27,28,29,30,31,32,33,34] as well as game animals such as Bezoar goats (*Capra aegagrus*) and Barbary sheep (*Ammotrgus* lervia) [35]. 

*T. gondii* infection is prevalent in different regions and its veterinary importance is significant because it causes abortion or congenital disease in small ruminants. Moreover, in small ruminants, infection not only results in significant reproductive losses but also has implications for public health since the consumption of infected meat can facilitate zoonotic transmission. For these reasons, the detection of *T. gondii* infection in sheep and goat populations is very important. Thus, this article presents the current status of the various detection possibilities of *T. gondii*, focusing on molecular methods including molecular targets and serological methods with the use of different forms of recombinant antigens. 

## 2. *T. gondii* Infection in Small Ruminants

In the 1950s, Hartley et al., for the first time, reported *T. gondii* as an important pathogen in sheep, which was detected in placental tissue from aborting sheep and fetal tissues [36,37]. Then, about 30 years later, other reports emerged of a similar disease in sheep occurring in Australia, UK, and Europe [38].

Sheep and goats become infected by ingesting food or water contaminated with oocysts excreted with the feces of definitive hosts or by transplacental transmission [5,39]. Since oocyst-contaminated pastures, fodder, and drinking water are regarded as potential sources of postnatal infection in small ruminants, these animals are considered biological indicators for the contamination of the environment with *T. gondii*. In addition, there are reports of a close relationship between the presence of cats on farms and the exposure of small ruminants to *T. gondii* [40,41,42]. What is more, the incidence of *T. gondii* infection is reported to increase with the age of the animals, especially for herds that are maintained in outdoor environments [40,42,43]. Thus, these data suggest that the most significant route of *T. gondii* transmission for small ruminants is via parasite oocysts found in the environment. 

The pattern of clinical disease and the pathology associated with *T. gondii* infection in sheep and goats are similar. Although most infections in small ruminants are asymptomatic, in the case of pregnant females, abortions, fetal mummification, stillbirths, and the birth of weak offspring may occur [44]. Furthermore, the stage of pregnancy in which transplacental transmission of the parasite occurs is very important in determining the clinical outcome. Initial *T. gondii* exposure and infection in pregnant females, when the fetal immune system is relatively immature, often results in fetal death. *T. gondii* infection occurring at mid-gestation can result in the birth of a stillborn or weak offspring accompanied by a small, mummified fetus, while infection in later gestation may result in the birth of live but infected offspring [39]. Furthermore, some studies have suggested that not only primary infection during a ewe’s pregnancy but also the recrudescence of an endogenous infection initiates transplacental transmission of the parasite and may also be an important route of congenital infection in sheep [45,46,47]. However, under conditions of endemic exposure, abortions are seen only in younger ewes. In addition to the time of maternal infection during pregnancy, the manifestation of symptoms and the severity of the disease depend on the immunological competence of the mother as well as the number and virulence of the parasites transmitted to the fetus. Lambs and goats that survive congenital infections usually grow regularly and therefore can be a source of *T. gondii* infection for humans. However, clinical signs may sometimes be present, and the parasite can be found in the organs and tissue of infected animals (mainly the liver, kidneys, and brain) [38]. 

## 3. Molecular Detection of *T. gondii*

Molecular techniques based on nucleic acid amplification can be used in addition to serology or in situations where employing serological methods is not possible, such as the diagnosis of young animals that have taken colostrum, or the detection of intrauterine *T. gondii* infection at early stages of gestation when the fetus is not immunocompetent [48]. In recent years, the diagnosis of toxoplasmosis in small ruminants based on the detection of parasitic DNA has been reported in multiple studies (Table 1).

The sensitivity of PCR-based techniques is related to the copy number of the amplified gene; therefore, highly repeated amino acid sequences can be used as molecular targets. Several assays employing multicopy sequences highly conserved among different strains of *T. gondii* have been developed. These PCR targets include the glycerol-3-phosphate dehydrogenase (*B1*) repetitive sequence (35 copies) [49]; the non-coding 529 bp repeated (REP529) sequence (200–300 copies) [50] and ribosomal DNA (110 copies); both of the genes encoding small subunit ribosomal RNA [51]; and the internal transcribed spacer (ITS1). Other single-copy genes such as *p30* (*sag1*) major surface antigen [52] and granule-dense antigen *gra7* [53] have also been reported as molecular targets.

In conventional PCR, the sensitivity varies depending on not only the molecular target but also the sample type, DNA extraction protocol, and amplification reaction conditions. To improve sensitivity and specificity, two consecutive PCR reactions (nested PCR) are commonly used. However, this approach increases the cost, time, and chances of contamination, and so it is therefore favorable to carry out relatively inexpensive and rapid single-tube nested PCR assays. Such an assay targeting the ITS1 region was developed by Hurtado et al. in 2001 [48] and applied for the diagnosis of *T. gondii*-induced abortion in fetal tissues from naturally aborted ewes. The technique makes use of two pairs of primers: external primers characterized by a higher melting temperature and internal primers with a lower melting temperature added in a larger concentration to suppress interference between the two amplification rounds. The detection limit was reported to be 1 pg (approximately 20 tachyzoites) in samples containing ovine tissue DNA [48].

Competitive PCR assays have been developed to quantify the number of *T. gondii* tachyzoites [50,54]. However, these methods are laborious and useful only within a narrow linear range of 2 to 3 logs of tachyzoites. Alternatively, a real-time PCR can be employed for quantitative analysis. Lin et al. in 2000 [55] described a highly sensitive and reproducible real-time quantitative PCR *B1* gene-specific TaqMan assay for the detection of *T. gondii* [55]. The major advantages of this approach are a long linear range over a minimum of 6 logs of DNA concentration as well as the ability to upscale the reaction to a high-throughput 96-well format and avoid post-PCR manipulations, such as gel electrophoresis [55]. 

Quantitative reverse transcriptase PCR (RT-PCR) has been reported as a feasible tool for assessing the viability of parasites [56,57], as mRNA is produced exclusively in metabolically active cells. RT-PCR assays for the screening of viable *T. gondii* have been described for targeting the SporoSAG gene expressed in sporulated oocysts coding a surface antigen glycoprotein [56,58]; the *act1* gene expressed in sporulated and unsporulated oocysts [58]; and SAG1 for the detection of tachyzoites [57]. It is important to note that PCR-based methods amplify the DNA of both live and dead cells and could lead to an overestimation of the amount of viable parasites [57].

**Table 1:** PCR-based assays used for *Toxoplasma gondii* detection in sheep and goat samples between 2017 and 2023.

Sample	Target Gene	Primers (5′-3′)	Product Size (bp)	Results	Reference, Publication Year
Muscle tissue of sheep and goats	*B1*	External primers Tg1: TGTTCTGTCCTATCGCAACG Tg2: ACGGATGCAGTTCCTTTCTG	580	*T. gondii* DNA was detected in 1.69% of sheep samples and 1.34% of goat samples.	[59], 2017
		Internal primers Tg3: TCTTCCCAGACGTGGATTTC Tg4: CTCGACAATACGCTGCTTGA	531		
Neck muscles of ewes and goats	*18S*	1A: AACCTGGTTGATCCTGCCAGT 564R: GGCACCAGACTTGCCCTC	600	DNA of *T. gondii* was detected in 33.3% of sheep and 32.5% of goats. The infection rate was significantly higher in sheep aged more than one year and goats aged less than three years in comparison to other ages.	[60], 2017
	*B1*	B22: AACGGGCGAGTAGCACCTGAGGAGA B23: TGGGTCTACGTCGATGGCATGACAAC	114		
Muscle tissue from ewes and ewe lambs	*18S*	1A: AACCTGGTTGATCCTGCCAGT 564R: GGCACCAGACTTGCCCTC	600	The percentage of *T. gondii* infection was 31% (54/174) and 32% (48/150) for animals from Sidi Bouzid and Beja, respectively. No significant difference in prevalence depending on age, breed and location was found.	[61], 2017
	*ITS1*	External primers NN1: CCTTTGAATCCCAAGCAAAACATGAG NN2 GCGAGCCAAGACATCCATTGCTGA	227		
		Internal primers Tg-NP1: GTGATAGTATCGAAAGGTAT Tg-NP2: ACTCTCTCTCAAATGTTCCT			
Sheep brains	*B1*	External primers Fext: GGAACTGCATCCGTTCATGAG Rext: TCTTTAAAGCGTTCGTGGTC	193	Parasitic DNA was detected in 26/140 (18.57%) samples.	[62], 2018
		Internal primers Fint: TGCATAGGTTGCAGTCACTG Rint: GGCGACCAATCTGCGAATACACC	97		
Ram semen	*18S*	1A: AACCTGGTTGATCCTGCCAGT 564R: GGCACCAGACTTGCCCTC	600	51.09% samples tested positive for *T. gondii* DNA.	[63], 2019
	*B1*	B22: AACGGGCGAGTAGCACCTGAGGAGA B23: TGGGTCTACGTCGATGGCATGACAAC	114		
Muscle tissue and diaphragm samples from sheep and goats	*B1*	External primers Fext: GGAACTGCATCCGTTCATGAG Rext: TCTTTAAAGCGTTCGTGGTC	193	No *T. gondii* DNA was detected in all 184 goat and sheep meat samples.	[64], 2020
		Internal primers Fint: TGCATAGGTTGCAGTCACTG Rint: GGCGACCAATCTGCGAATACACC	96		
Muscle tissue of seropositive sheep and goats	*ITS1*	External primers NN1: CCTTTGAATCCCAAGCAAAACATGAG NN2 GCGAGCCAAGACATCCATTGCTGA	227	For goats, similar prevalence was found for both young and adult animals (6.9% and 4.5%, respectively); a significant difference was reported between adult sheep and lambs (38.2% and 1%, respectively).	[65], 2020
		Internal primers Tg-NP1: GTGATAGTATCGAAAGGTAT Tg-NP2: ACTCTCTCTCAAATGTTCCT			
Muscle tissue of sheep and goats	*B1*	External primers TOXO1: GGAACTGCATCCGTTCATGAG TOXO2: TCTTTAAAGCGTTCGTGGTC	98	Parasite DNA was detected in 9.84% of sheep and 10.73% of goats. Meat from rural areas had a significantly higher *T. gondii* prevalence than that obtained from supermarkets.	[66], 2020
		Internal primers TOXO4: TGCATAGGTTGCAGTCACTG TOXO2: TCTTTAAAGCGTTCGTGGTC			
Blood of female sheep and goats that aborted	*B1*	JW63: GCACCTTTCGGACCTCAACCG JW62: TTCTCGCCTCATTTCTGGGTCTAC	286	DNA of *T. gondii* was detected in 35.24% samples of sheep and 18.68% of goats. Prevalence was higher in sheep aged over 1 year. No significant difference in relation to age in goats. Female sheep that aborted between 1 and 60 days of gestation were more often infected then females that aborted between days 61 and 120. The opposite was reported for goats.	[67], 2020
Lamb mincemeat purchased from a large supermarket	*B1*	Real-time PCR TOXO-F: TCCCCTCTGCTGGCGAAAAGT TOXO-R: AGCGTTCGTGGTCAACTATCGATTG	98	43% of lamb mincemeat was contaminated with *T. gondii*.	[68], 2020
		Nested PCR F: CGAAAAGTGAAATTCATGAG R: CTATCGATTGCAGGCGACC	66		
Heart, liver, and meat tissues of sheep and goats	*B1*	F: GAGACCGCGGAGCCGAAGTGC R: CCTCCTCCTCCCTTCGTCCAAG	469	17.3%, 22%, and 32% of liver, meat, and heart samples in sheep, and 16%, 17.3%, and 24% of liver, meat, and heart samples in goats, respectively, showed positive PCR results.	[69], 2021
Ovary, horns, body of the uterus, and vagina of chronically infected ewes	*18S*	1A: AACCTGGTTGATCCTGCCAGT 564R: GGCACCAGACTTGCCCTC	600	95.2% of ewes had at least one infected genital part. A significantly higher parasitic prevalence was found in the ovaries and vagina of older animals.	[70], 2021
	*B1*	B22: AACGGGCGAGTAGCACCTGAGGAGA B23: TGGGTCTACGTCGATGGCATGACAAC	114		
Liver or diaphragm tissue of goats and sheep	*B1*	External primers Fext: GGAACTGCATCCGTTCATGAG Rext: TCTTTAAAGCGTTCGTGGTC	193	*T. gondii* detected in 14.4% (13/90) of sheep and 8.8% (8/90) of goats. No statistically significant difference between infection rate and age or sample type. However, significantly more males (19.5%) than females (3.4%) were found to be infected.	[71], 2021
		Internal primers Fint: TGCATAGGTTGCAGTCACTG Rint: GGCGACCAATCTGCGAATACACC	96		
Goat blood	*ITS1*	F: AGTTTAGGAAGCAATCTGAAAGCACATC R: GATTTGCATTCAAGAAGCGTGATAGTAT	302	*T. gondii* DNA was detected in 48/898 (5.3%) goats. Blood cell count and serum creatine was affected in *T. gondii*-positive animals.	[72], 2022
Sheep and goat blood	*B1*	External primers Fext: GGAACTGCATCCGTTCATGAG Rext: TCTTTAAAGCGTTCGTGGTC	193	Corresponding results of PCR assay and ELISA. B1-PCR products more intense then single P30-PCR products.	[73], 2022
		Internal primers Fint: TGCATAGGTTGCAGTCACTG Rint: GGCGACCAATCTGCGAATACACC	97		
	*P30*	P30F: TTGCCGCGCCCACACTGATG P30R: CGCGACACAAGCTGCGATAG	914		
Tissues of seropositive sheep (brain, heart, lungs, kidneys, liver, and diaphragm)	*P43*	External primers Fext: CAACTCTCACCATTCCACCC Rext: GCGCGTTGTTAGACAAGACA	225	DNA of the parasite was detected in 60% of animals. Pairs of tissue of lungs and heart, lungs and diaphragm or heart and diaphragm could be employed for successful molecular detection of *T. gondii* in sheep.	[74], 2022
		Internal primers Fint: TCTTGTCGGGTGTTCACTCA Rint: CACAAGGAGACCGAGAAGGA			
Brain and heart from sheep abortions	*REP529*	TOX4: CGCTGCAGGGAGGAAGACGAAAGTTG TOX5: CGCTGCAGACACAGTGCATCTGGATT		*T. gondii* DNA was detected in 11.8% (9/76) of sheep abortions. Both brain and heart samples were positive in PCR.	[75], 2023
Brains of mice bioassayed with diaphragm tissue from seropositive lambs			529	Viable *T. gondii* was isolated from 32.14% lambs by microscopic examination for brain cysts. PCR confirmed these results.	
Sheep hearts	*ITS1*	External primers NN1: CCTTTGAATCCCAAGCAAAACATGAG NN2 GCGAGCCAAGACATCCATTGCTGA	227	The molecular detection of *T. gondii* was 7.3%, while seroprevalence was 26.1%. Nested PCR increased the sensitivity in comparison to conventional PCR.	[76], 2023
		Internal primers Tg-NP1: GTGATAGTATCGAAAGGTAT Tg-NP2: ACTCTCTCTCAAATGTTCCT			
Goat blood	*B1*	External primers Tg1: TGTTCTGTCCTATCGCAACG Tg2: ACGGATGCAGTTCCTTTCTG	580	Prevalence of infection was 14.8% (25/169)	[77], 2023
		Internal primers Tg3: TCTTCCCAGACGTGGATTTC Tg4: CTCGACAATACGCTGCTTGA	531		

## 4. *T. gondii* DNA Detection in Milk

Human toxoplasmosis has been linked epidemiologically to drinking raw goat and sheep milk in many studies over the last 40 years [78,79,80,81,82]. In 1990, Patton et al. [83] found *T. gondii* antibodies in 80% of goats from a farm in Tennessee. The parasite was isolated from the brain of a child who died after the mother drank unpasteurized goats’ milk from the farm and developed toxoplasmosis while pregnant [83]. More recently, a study performed by the Brazilian Ministry of Health found that over the span of three years, milk and milk products carried causative pathogens in 337 out of over 8000 foodborne disease outbreaks [84].

Many studies have confirmed the presence of *T. gondii* DNA in the milk of sheep (Table 2) and goats (Table 3). No correlation has been reported between the results of PCR-based DNA detection and the serological status of the lactating animals [85,86], as the excretion of *T. gondii* in milk is irregular and may be dependent on many factors such as the parasite strain and diurnal cycle [87]. On the other hand, research on both goats and sheep has shown a kappa coefficient of 1 for the association between the results of PCR on blood samples and the results of PCR on milk samples, meaning that 100% of animals testing positive for PCR on blood were excreting *T. gondii* DNA in their milk [88,89]. It is important to note that the opposite correlation is not observed, as not all animals testing positive for PCR on milk samples had parasite DNA found in their blood.

Molecular diagnostic tools based on PCR are highly dependent on the quality of DNA samples. Extracting nucleic acids from milk is hindered by a high fat content which does not allow the use of many commonly used reagents. In 2017, Vismarra et al. [90] assessed three pre-treatment protocols for DNA extraction from sheep milk spiked with tachyzoites. Protocol I consisted only of a series of centrifugations and required at least an hour. Protocol II aimed to remove caseins prior to DNA extraction and took approximately 20 min. In protocol III, milk was added to a lysis solution with proteinase K and incubated overnight. The authors concluded that there is no significant difference in terms of sensitivity depending on the pre-treatment method and therefore chose the least time-consuming protocol, protocol II, for the analysis of 21 field samples from sheep of Southern Italian farms. The occurrence of parasitic DNA was determined by real-time PCR targeting the Repeat region of 529 bp and resulted in one positive sample [90].

The demonstration of DNA in milk does not equate to the viability of the parasite in the sample. In 2014, Dubey et al. [87] showed by mouse bioassay that viable *T. gondii* can be found in the unpasteurized milk of experimentally infected goats and that parasite excretion is intermittent. The authors found no correlation between the bioassay results and the detection of *T. gondii* DNA by PCR [87].

The most likely cause of *T. gondii* excretion in milk is the infection of milk-secreting cells and their subsequent shedding, making the tachyzoite the logical stage that is expelled [87]. This stage of the parasite is typically very sensitive to acidic pH and, therefore, is considered unlikely to survive stomach passage. However, studies have shown that tachyzoites retained infectivity in simulated gastric fluid of pH 5.0 and 6.0 for at least 90 min. The pH of the stomach ranges from 1 to 3 and rises quickly to about 7 after food ingestion. As a result, milk-ingested tachyzoites encounter pH levels that are higher than the acidic contents of the stomach. This makes it more likely that some tachyzoites may penetrate the stomach mucosa or reach the alkaline duodenum and infect cells in the intestinal tract [91]. Alternatively, infection by tachyzoites might occur by penetration of the oropharyngeal mucosa [78]. 

Consumption of milk contaminated with *T. gondii* presents a viable risk of acquiring toxoplasmosis, as tachyzoites were shown to survive for several days in milk and conserve their infectivity for up to 30 min in milk media heated to 37 °C. [92]. It is therefore recommended that milk should be pasteurized prior to human consumption to eliminate any potentially infectious tachyzoites.

The presence and viability of *T. gondii* in cheese made from raw milk has also been studied. In 2020, Ranucci et al. [57] concluded by RT-PCR that cheese made from naturally contaminated ewe milk does not contain metabolically active tachyzoites; however, there were no data regarding the amount of *T. gondii* in the milk used for cheesemaking. Dubey et al. [87] reported the survival of *T. gondii* in cheese made via the cold-enzyme treatment of raw goat milk intentionally contaminated with tachyzoites by subcutaneously inoculating mice. The study also found that cheese made with tachyzoite-spiked milk is not infectious to mice via the oral route.

**Table 2:** Studies reporting the detection of *Toxoplasma gondii* DNA in sheep milk.

Detection Method	Target Gene	Results	Reference, Publication Year
PCR	REP529	Seven milk samples from five seropositive sheep tested positive. Authors stated that the peripartum period may lead to the recirculation of T. gondii tachyzoites which can be excreted in milk.	[93], 2011
PCR	B1	1/27 (3.7%) milk samples tested positive	[94], 2011
Real-time PCR	TGR1E	*T. gondii* DNA detected in 7/25 (28%) milk samples from IgM+ sheep and 2/55 (3.64%) samples from IgM-sheep.	[89], 2014
PCR Real-time PCR	REP529	1/21 (4.76%) milk samples positive in both one-step PCR and real-time PCR	[90], 2017
Nested PCR	ITS1 and B1	1/58 (1.72%) milk samples showed presence of *T. gondii* DNA.	[95], 2019
LAMP RT-PCR	SAG1	16/16 milk samples positive in both methods. LAMP demonstrated the presence of *T. gondii.* DNA and RT-PCR assessed parasite viability.	[57], 2020
PCR	B1	5/45 (11.11%) milk samples positive for parasitic DNA.	[96], 2022

**Table 3:** Studies reporting the detection of *Toxoplasma gondii* DNA in goat milk.

Detection Method	Target Gene	Results	Reference, Publication Year
Nested PCR	unknown	13% of milk samples from seropositive goats tested positive. 100% agreement between results for blood and milk samples.	[88], 2013
PCR	REP529	*T. gondii* DNA was detected in 15/248 (6.05%) of milk samples. 5/15 positive samples were from seropositive goats.	[97], 2015
PCR	REP529	2.69% (5/186) of goat milk samples tested positive by PCR. None of the samples came from IgG+ animals.	[84], 2015
Nested PCR	ITS1	7.8% of milk samples tested positive. No correlation between DNA in milk and seroprevalence.	[86], 2016
Real-time PCR Nested PCR	B1	65% of milk samples were positive in real-time PCR and 43% in nested PCR. Some positive samples were obtained from IgG-goats.	[85], 2017
PCR	B1	1/29 milk samples (3.4%) tested positive.	[94], 2017
Nested PCR	ITS1	Excretion of parasite DNA was intermittent. Highest DNA concentration was found in the second fortnight and at the end of lactation. No milk samples tested positive in the first fortnight of lactation.	[98], 2019
Nested PCR	B1	11 samples (5.5%) of goat milk tested positive for *T. gondii* DNA. No significant relationship between geographical area and milk infection.	[99], 2021
PCR	B1	Molecular prevalence of *T. gondii* in milk 20%.	[96], 2022

## 5. Serological Detection of *T. gondii*

Serological tests are essential tools for recognizing *T. gondii* exposure that are widely used in epidemiological studies among different populations of animals. This indirect detection plays a crucial role in the diagnosis of toxoplasmosis, especially when a specific clinical sign is absent in an immunocompetent host of the parasite. Usually, *Toxoplasma* serology relies on the detection of both IgG and IgM antibodies. The positivity for specific IgM is a marker of acute infection due to the occurrence of this antibody within days to a couple of weeks. Nevertheless, the detection of IgM antibodies bears limitations in estimating the time of *T. gondii* infection in humans or animals since low IgM titers may persist long after the acute phase of the disease [100,101,102,103,104]. Thus, the identification of specific IgG is a key parameter to confirm primary *T. gondii* infection or to indicate past infection [104]. The level of anti-*T. gondii* IgG antibodies peaks typically within 1–2 months after infection and declines at various rates. Moreover, these antibodies can have lifelong persistence in residual titers. However, the detection of this class of antibodies alone cannot differentiate the early phase from the chronic phase of *T. gondii* infection. For the differentiation of acute from chronic infection, the IgG avidity test is commonly used in the serodiagnosis of toxoplasmosis in humans [105]. IgG antibodies produced in the recent primary *T. gondii* infection are of low avidity while IgG antibodies with high avidity are detected in the chronic phase of infection. The IgG avidity assay has also been used in research to discriminate between acute and chronic *T. gondii* infection in sheep [106,107].

Many different IgG assays are available worldwide, either manual or fully automated, and are based on various detection methods including agglutination assays, Western blot assays, immunofluorescence assays, enzyme immunoassays, and rapid diagnostic tests (RDTs) based on immunochromatography. These assays have variable performances and thresholds of detection; therefore, it is sometimes difficult to compare their results. Among various serodetection tests, ELISA has been accepted as the most practical test for the diagnosis of toxoplasmosis and, for this reason, is the most frequently used. Furthermore, in the serodiagnosis of *T. gondii* infection, the specificity and sensitivity of anti-*Toxoplasma* IgG assays are crucial. These test parameters mainly depend on the level of individual classes of antibodies produced during infection and the type of antigen used in the serological assay. Most commercial serological kits use native antigens of parasites prepared from tachyzoites grown in mice and/or tissue culture in vitro. These kinds of antigens obtained from tachyzoites may contain various sources of nonparasitic material from eukaryotic host cells or culture media. For this reason, serological assays based on *Toxoplasma* lysate antigens (TLA) are difficult to standardize and frequently provide insufficient specificity. Furthermore, the production of TLA preparation is considered expensive, time-consuming, and labor-intensive. The development of molecular biology and genetic engineering methods in the second half of the 20th century made it possible to develop new diagnostic tools—biotechnologically produced recombinant antigens. These preparations of antigens are usually available in a pure form; thus, the use of such antigen preparations in diagnostic tests has many advantages, such as precise determination of the antigenic composition, the possibility of using more than one antigen in the test, and much easier standardization of the method. For these reasons, many different recombinant antigens of *T. gondii* as single proteins, mixtures of various antigens, or chimeric proteins have already been successfully used for the serodiagnosis of toxoplasmosis in humans [104,108] and different populations of animals [109]. Numerous attempts have been made worldwide to produce recombinant *T. gondii* antigens, particularly using the prokaryotic expression systems based mainly on *Escherichia coli* cells. Most of the existing papers concern the diagnostic use of various forms of recombinant proteins for the detection of specific antibodies in human sera. However, in the literature there are also reports regarding the use of such antigenic preparations in serological detection in small ruminants (Table 4 and Table 5). Among the various parasitic proteins, cell surface antigens and proteins from secretory organelles such as micronemes, rhoptries, and dense granules have been in focus for their potential as diagnostic tools. The first information about the use of recombinant proteins in the serodiagnosis of ovine toxoplasmosis appeared in the literature over 30 years ago when in 1992 Tenter at al. [110] showed the reactivity in IgG ELISA of two H4 and H11 recombinant proteins, expressed in *E. coli* as glutathione-S-transferase (GST) fusion polypeptides with specific ovine antibodies. Promising results in the detection of *T. gondii*-specific antibodies from canine and ovine serum samples were also obtained for several other single recombinant antigens obtained in a bacterial expression system (Table 4). However, the sensitivity of IgG ELISAs, which were based on single recombinant antigenic proteins, varied widely. The best results were obtained by Holec-Gąsior et al., in 2014 [111] for three recombinant proteins, GRA1, P22 and ROP1, which recognized specific antibodies from sheep sera with 100% specificity and 100% sensitivity for GRA1 and ROP1 and 98.9% for P22. Furthermore, in some cases, *T. gondii* recombinant antigens were used in IgG ELISAs in various combinations consisting of two or three proteins (Table 5). The use of an appropriately selected mixture of recombinant antigens enhanced the sensitivity of an antibody-based assay; for example, a mixture of the three antigens listed above resulted in 100% sensitivity and specificity in the IgG ELISA [111]. The same result was obtained in 2015 by Ferra et al. [112] for the mixture of three recombinant proteins: SAG2 + GRA1 + ROP1. In addition, in recent years, serological research has started to use the so-called multivalent chimeric proteins (Table 5). These antigenic preparations are a new kind of diagnostic tool that have been successfully used to detect specific antibodies in human sera [108,113,114,115,116,117]. A single recombinant multivalent chimeric protein contains different immunoreactive epitopes from various *T. gondii* antigens which have been properly selected. For this reason, chimeric preparation can replace a mixture of several recombinant antigens. What is more, the combination of carefully selected epitopes from proteins of different stages of the *T. gondii* life cycle is an optimal strategy for overcoming the antigen complexity of the parasite [108]. Therefore, the chimeric protein may be a more immunodominant antigen than the original antigens. In 2015, Ferra et al. [112] evaluated the usefulness of five *T. gondii* recombinant chimeric proteins composed of three different well-characterized parasite proteins for the detection of specific IgG antibodies in sera from naturally infected sheep. These multivalent chimeric proteins were generally more reactive than mixtures of three recombinant antigens. The most effective combinations for the diagnosis of ovine toxoplasmosis were three tested chimeric proteins (SAG1_L_-MIC1-MAG1, SAG2-GRA1-ROP1_S_, and SAG2-GRA1-ROP1) which showed 100% sensitivity and specificity in the IgG ELISA. Furthermore, in 2019 Holec-Gąsior et al. [118] for the first time described the reactivity of four tetravalent chimeric proteins (AMA1_N_-SAG2-GRA1-ROP1, AMA1_C_-SAG2-GRA1-ROP1, AMA1-SAG2-GRA1-ROP1, and SAG2-GRA1-ROP1-GRA2) with specific IgG antibodies from the sera of small ruminants in IgG ELISA. All tested chimeric proteins were characterized by high specificity (between 96.39% to 100%), while the highest sensitivity was observed in the IgG ELISA test based on AMA1-SAG2-GRA1-ROP1 (Table 5). Thus, studies conducted in recent years have shown that recombinant chimeric proteins can be successfully used to diagnose *T. gondii* infection in small ruminants and can replace the commonly used TLA. 

## 6. Summary and Conclusions

Toxoplasmosis poses a serious risk to immunocompromised individuals and congenitally infected fetuses; it is, therefore, crucial to monitor and properly diagnose this disease. Consuming raw or undercooked meat containing tissue cysts has been found to be the most important risk factor linked to human toxoplasmosis. Small ruminant meat and cured meat products are a major source of this disease, especially in countries where goat meat and mutton are regularly consumed, as ovine and caprine hosts are highly prone to *T. gondii* infection. What is more, tachyzoites are excreted intermittently throughout lactation in sheep and goat milk and the consumption of unpasteurized milk or dairy has been linked to toxoplasmosis outbreaks. Toxoplasmosis is also of veterinary importance as it leads to significant reproductive losses in animals. For all of the above-mentioned reasons, the sensitive and reliable detection of *T. gondii* in the tissues and milk of small ruminants is imperative. Furthermore, the great importance of *T. gondii* as a causative agent of zoonosis indicates the need for extensive epidemiological studies on the population of animals that can be used as a source of food. Thus, it is very important to carry out extensive diagnostic research in the population of small ruminants worldwide, not only because of the potential elimination of the source of transmission of the parasite but also to aid in the selection of healthy animals that can be vaccinated with the Ovilis^TM^ Toxovax vaccine that is approved in some countries. However, it should be noted that this is the only available registered vaccine for sheep, which comprises live attenuated *T. gondii* S48 strain tachyzoites, has limited use, and can only reduce the incidence of abortion and neonatal mortality due to congenital infection [127,128]. Therefore, the vaccination of these animals used for meat seems a promising prevention strategy, but it is still in the experimental phase and needs further development. In addition, there are some reports in the literature that suggest that the meat of animals intended for consumption should be properly tested and marked as ‘*Toxoplasma*-free meat’ [18,129]. In 2007, the Biological Hazard Panel of the European Food Safety Authority recommended that *Toxoplasma* monitoring programs should be initiated in the preharvest sector on sheep, goats, pigs, and game [2]. In 2009, Kijlstra and Jongert [129] stated that a special pre-harvest monitoring program could aim to select *T. gondii*-seronegative animals for sale as *Toxoplasma*-free meat, whereas meat with positive tests could be subjected to thermal pre-treatment or freezing and then sold as meat safe from *T. gondii*. Therefore, the detection of *T. gondii* infection in small ruminants with the use of different diagnostic tools that would allow the rapid and cheap testing of numerous samples of biological material from sheep and goats is being extensively developed. In this article, we focused on the various detection possibilities for *T. gondii*, such as molecular and serological methods.

Several molecular detection methods have been developed for the detection of parasitic DNA, which can be carried out in conjunction with serodiagnosis or in situations where serological methods cannot be employed, such as the diagnosis of not-yet immunocompetent fetuses at an early stage of gestation or young animals that have taken colostrum [48]. PCR-based techniques make use of amino acid sequences that are repeated and highly conserved among different *T. gondii* strains. To increase the sensitivity of conventional PCR, most studies use a nested technique, but this approach is susceptible to contamination; therefore, single-tube nested PCR assays were developed. To quantify the number of tachyzoites, competitive PCR assays have been described; however, real-time PCR is reported to be a better alternative for quantitative analysis. It is important to note that the detection of parasitic DNA does not equate to the viability of the parasite in the sample; therefore, RT-PCR has been reported as an alternative to bioassays in assessing the viability of *T. gondii.* Molecular methods have been successfully employed in detecting tachyzoites in ruminant milk. However, no correlation has been found between the results of PCR and the serological status of the lactating animal, whereas 100% of animals testing positive for PCR on blood also tested positive for *T. gondii* DNA in their milk. What is more, in 2014, Dubey et al. [87] reported no relationship between the detection of parasitic DNA and mouse bioassay results for the milk of experimentally infected goats. The excretion of tachyzoites by lactating ruminants is still puzzling and requires further investigation.

Serological tests are the most widely used *T. gondii* detection method in epidemiological studies, specifically ELISA. Most commercially available tests utilize TLA, which leads to difficulty in standardization and insufficient specificity. Biotechnologically produced recombinant proteins have been proposed as an alternative diagnostic tool, as they are characterized by a precisely known composition, easier standardization, and the possibility of using appropriately selected mixtures of recombinant antigens to further increase the sensitivity of serological assays. Many studies have reported the diagnostic utility of recombinant parasitic proteins, mainly focusing on cell surface antigens and proteins from secretory organelles such as micronemes, rhoptries, and dense granules. Analysis of the results reported in those publications has shown that different cloning strategies and variations in the recombinant protein purification methods resulted in different levels of sensitivity and specificity being obtained in the diagnostic tests conducted in various laboratories working with the same antigens: for example, the results obtained in IgG ELISAs with the use of SAG1 or SAG2 recombinant antigens [105,113,115,116,118,120]. In addition, for some studies concerning the application of the same recombinant antigens in IgG ELISAs, it is difficult to compare the obtained results because the criteria for negative and positive test results vary among researchers. Moreover, current serological research has employed multivalent recombinant proteins for the detection of specific ani-*T. gondii* antibodies in sera. This approach of combining several immunodominant protein fragments into one antigen can be used as an alternative to protein mixtures and offers higher reproducibility, in addition to enabling the antigen complexity of the parasite related to different life stages of *T. gondii* to be overcome. To the best of our knowledge, B-cell epitope mapping appears to be a key step in the rational design of chimeric proteins. It enables the identification of highly specific epitopes, which allows the selection of appropriate fragments for the construction of multivalent proteins. Peptide microarrays are the most popular method for mapping linear B-cell epitopes, which is quick and low-cost as they allow the analysis of thousands of peptides simultaneously. This approach allows for the determination of sequences recognized by specific antibodies in a complete antigen sequence. Therefore, it is currently the basis of many studies aimed at identifying new proteins with diagnostic or immunoprotective utility [130]. Furthermore, the search for new, more-effective antigenic proteins of the parasite that can be used as serological diagnostic tools in the future is also important. For this purpose, bioinformatics has become very helpful, being widely used to predict protein structures, functions, and other biological characteristics [131].

Although mouse bioassays are considered the gold standard for detecting parasitic infection in tissues, clinical diagnosis is carried out by serology and/or molecular methods, as bioassays are time-consuming, costly, cause animal distress, and are not suited for the screening of many samples. PCR-based techniques are less sensitive than bioassays due to the uneven distribution of tissue cysts in host tissue [4]; therefore, new molecular targets and novel molecular procedures continue to be a focus of development towards increasing test sensitivity. Methods based on DNA amplification have proven effective in detecting *T. gondii* infection in a multitude of goat and sheep tissues: muscle, blood, brain, semen, diaphragm, heart, liver, genitals, and kidneys, as well as the milk of lactating animals. In summary, research to date has shown the potential diagnostic utility of *T. gondii* recombinant antigens in various forms of protein preparation for the detection of parasitic infection in small ruminants’ sera. Certainly, the use of recombinant proteins in the serodiagnosis of *T. gondii* infection would be highly beneficial in improving the standardization of assays and reducing their production costs. Additionally, it is possible to use these antigenic preparations in other diagnostic assays, such as the rapid lateral flow test based on the immunochromatographic method or the latex agglutination test. In our opinion, this could additionally improve and facilitate serological examinations in small ruminant populations.

A variety of diagnostic methods have been employed for the detection of *T. gondii* infection in small ruminants. For most valid results, it is recommended to carry out a serological assay followed by PCR analysis as the presence and level of specific antibodies is not always correlated with the occurrence of parasitic DNA in tested samples. Even though progress continues to be made in toxoplasmosis diagnosis, there are still advancements in both molecular and serological methods that should be studied and developed. It is also important to focus on possible false-positive results caused by the genetic similarity of other Apicomplexa-type parasites. *Neospora caninum* is the most closely taxonomically related to *T. gondii* and has been proven to infect small ruminants [132]. Moreover, both infections present similar symptoms. Reliable serodiagnosis must take into consideration possible antibody cross-reactivity and any new recombinant antigens should be determined as species-specific in order to show diagnostic utility. 

## Figures and Tables

**Table 4:** Single *Toxoplasma gondii* recombinant antigens used for the detection of specific antibodies in small ruminants’ sera.

Recombinant Antigen Used in Test	Serologic Test ^1^	No. of Examined Sera	Results	Reference, Publication Year
H4 H11	IgG ELISA	26 (sheep)	Compared with an ELISA based on traditional parasite antigen, the ELISA for sheep sera had a sensitivity of 79% and 43% for H4 and H11 recombinant antigens, respectively, while the specificity was 100% for both proteins.	[110], 1992
H11	IgG ELISA	92 (sheep)	Compared with an ELISA based on traditional parasite antigen, the H11-ELISA for sheep sera had a sensitivity of 34% and the specificity was 89%.	[106], 2003
surface antigen 1—SAG1 granule dense antigen 7—GRA7	IgG ELISA	56 (goat)	The sensitivity of the SAG1-ELISA and GRA7-ELISA was 83.3%% and 80.0%, respectively, while the specificity was 88.4% for both tests.	[119], 2008
matrix antigen 1—MAG1	IgG ELISA	175 (sheep)	24% of sheep serum samples were detected as positive for *T. gondii*-specific antibodies. Results of rTgMAG1-ELISA were compared to LAT test, and the same result was obtained for both LAT and ELISA for 27 (15.4%) sera.	[120], 2010
19 proteins: granule dense antigens—GRA1; GRA2ex2; GRA4; GRA5; GRA6; GRA9; surface antigens—SAG1; P22; SAG4; BSR4; rhoptry antigens—ROP1; ROP9; microneme antigens—MIC1ex2; MIC1ex34; MIC3; matrix antigen 1—MAG1; bradyzoite antigen 1—BAG1; lactate dehydrogenase—LDH1; LDH2	IgG ELISA	108 (sheep)	Preliminary evaluation of 19 single recombinant antigens in IgG ELISA with 2 positive and 2 negative serum samples. 8 antigens (GRA1, GRA9, SAG1, P22, SAG4, MIC1ex2, MIC3, and ROP1) were selected for further analysis with a pool of 108 serum samples. 3 antigens (GRA1, P22, ROP1) were selected with sensitivity (98.9–100%) and specificity (100%).	[111], 2014
surface antigen 2—SAG2	IgG ELISA	63 (goat) 60 (sheep)	50% sheep serum samples and 41.26% goat samples were detected as positive for *T. gondii*-specific antibodies. Compared to IFAT, the sensitivity of the rec-SAG2-ELISA was 82.1% and 81.3% for goats and sheep, respectively. Compared to IFAT, the specificity of the rec-SAG2-ELISA was 91.4% and 85.7% for goats and sheep, respectively.	[121], 2015
surface antigen 2—SAG2	IgG ELISA	249 (goat) 610 (sheep)	20% sheep serum samples and 12.9% goat samples were detected as positive for *T. gondii*-specific antibodies. Serum samples were not tested by any commercial assay.	[122], 2016
granule dense antigen 7—GRA7	IgG ELISA	94 (goat) 111 (sheep)	51.4% sheep serum samples and 39.4% goat samples were detected as positive for *T. gondii*-specific antibodies. Results of TgGRA7-ELISA were compared to LAT test, and the same result was obtained for both LAT and ELISA for 27 (28.7%) and 43 (38.7%) goat and sheep sera, respectively.	[123], 2016
surface antigen 1—SAG1	IgG ELISA	445 (sheep)	42.5% sheep serum samples were detected as positive for * T. gondii * -specific antibodies. Compared to IFAT, the sensitivity and specificity of the rSAG1-ELISA were 92.7% and 90.7%, respectively.	[124], 2018
granule dense antigen 8—GRA8	IgG ELISA	306 (goat)	15.40% of goat serum samples were positive for IgG *T. gondii*-specific antibodies.	[125], 2021
surface antigen 1—SAG1 granule dense antigen 7—GRA7 bradyzoite antigen 1—BAG1	IgG ELISA IgM ELISA	904 (Tibetan sheep)	18%, 9.7% and 4.1% of sheep serum samples were detected as positive for IgG * T. gondii * -specific antibodies in rSAG1-ELISA, rGRA7-ELISA and rBAG1-ELISA, respectively. 1.4%, 0.7% and 1.2% of sheep serum samples were detected as positive for IgM * T. gondii * -specific antibodies in rSAG1-ELISA, rGRA7-ELISA and rBAG1-ELISA, respectively.	[126], 2022

^1^ ELISA—enzyme-linked immunosorbent assay.

**Table 5:** Mixture of *Toxoplasma gondii* recombinant antigens and chimeric, multiepitope recombinant proteins used for the detection of specific antibodies in small ruminants’ sera.

Recombinant Antigens Used in Test	Serologic Test ^1^	No. of Examined Sera	Results	Reference, Publication Year
Mixture: SAG1 + GRA7	IgG ELISA	56 (goat)	The sensitivity and specificity of the IgG ELISA were 88.6% and 88.4%, respectively.	[119], 2008
Mixtures: M1: GRA1 + ROP1; M2: GRA1 + P22; M3: P22 + ROP1; M4: GRA1 + P22 + ROP1	IgG ELISA	236 (sheep)	Mixtures were initially tested with a pool of 108 serum samples. The sensitivity of all IgG ELISAs was equal to 100%, while the specificity was varied and was 100% for M1-ELISA and M4-ELISA and 95% for M2-ELISA and M3-ELISA. The M4 mixture showed the highest reactivity and was tested with a new pool of 128 sera. 100% sheep serum samples were detected as positive for *T. gondii*-specific antibodies. The specificity of M4-ELISA was also 100%.	[111], 2014
Mixtures: M1: SAG1 + MIC1 + MAG1; M2: SAG2 + GRA1 + ROP1; M3: GRA1 + GRA2 + GRA6	IgG ELISA	191 (sheep)	The sensitivity of M1-ELISA, M2-ELISA and M3-ELISA was 77.9%, 100% and 92.1%, respectively. The specificity of M1-ELISA, M2-ELISA and M3-ELISA was 92.2%, 100% and 100%, respectively.	[112], 2015
Chimeric proteins: MIC1-MAG1-SAG1_S_; SAG1_L_-MIC1-MAG1; SAG2-GRA1-ROP1_S_; SAG2-GRA1-ROP1_L_; GRA1-GRA2-GRA6	IgG ELISA	191 (sheep)	The specificity of all IgG ELISAs was equal to 100%. The sensitivity of three IgG ELISAs with the use of SAG1_L_-MIC1-MAG1, SAG2-GRA1-ROP1_S_, SAG2-GRA1-ROP1 was 100%, while the sensitivity of two IgG ELISAs based on MIC1-MAG1-SAG1_S_ and GRA1-GRA2-GRA6 was 97.9% and 92.1%, respectively.	[112], 2015
Chimeric proteins: AMA1N-SAG2-GRA1-ROP1; AMA1C-SAG2-GRA1-ROP1; AMA1-SAG2-GRA1-ROP1; SAG2-GRA1-ROP1-GRA2	IgG ELISA	86 (goat) 90 (sheep)	The sensitivity of the IgG ELISA based on AMA1_N_-SAG2-GRA1-ROP1 was 88.9% and 97.9% for goats and sheep, respectively. The sensitivity of the IgG ELISA based on AMA1_C_-SAG2-GRA1-ROP1 was 95.6% and 95.8% for goats and sheep, respectively. The sensitivity of the IgG ELISA based on AMA1-SAG2-GRA1-ROP1was 95.6% and 97.9% for goats and sheep, respectively. The sensitivity of the IgG ELISA based on SAG2-GRA1-ROP1-GRA2 was 57.8% and 97.9% for goats and sheep, respectively. The specificity of all IgG ELISA tests varied from 95.1% to 100%.	[118], 2019

^1^ ELISA—enzyme-linked immunosorbent assay.

## Data Availability

No new data was created or analyzed in this study. Data sharing is not applicable to this article.
